# Imaging of Malar Silastic Implant Complications

**DOI:** 10.7759/cureus.34874

**Published:** 2023-02-11

**Authors:** Vincent F Carfagno, Zahivette Lopez Ramos, Eleazar Fierro, Lazarus Gutierrez, Imtiaz Ahmed

**Affiliations:** 1 MSIII, Midwestern University Arizona College of Osteopathic Medicine, Glendale, USA; 2 MSIV, Universidad Autonoma Guadalajara School of Medicine, Jalisco, MEX; 3 Radiology, Tempe St. Luke's Hospital, Tempe, USA

**Keywords:** intrasinus migration, malar implant, facial cosmetic surgery, silicone implant, cosmetic surgery, radiology

## Abstract

Facial cosmetic implants are utilized for definition enhancements in the malar, mandibular, and nasal regions. Though these implants are safe in the majority of patients, notable complications such as implant malpositioning may be seen. More rare but serious complications such as infection, abscess, and intrasinus migration may also occur, such as in this case reported on a 69-year-old female with a history of bilateral malar silicone implants. Imaging findings on this patient, whom initially presented with complaints of erythema and edema in the left malar region, were notable for edema and soft tissue signs of infection around a well-visualized crescent shaped maxillary implant. Penetration of the implant into the left maxillary sinus was also noted. Diagnostic imaging played a key role in determining the cause and severity of this patient’s condition. Thus, the case reported is with an aim to familiarize radiologists with identifying and interpreting the complications of malar cosmetic implants on diagnostic imaging.

## Introduction

Malar augmentation consists of adding volume to either the malar or submalar space to elevate the surrounding subcutaneous tissues [1]. Current techniques commonly involve the implantation of silastic shell implants due to their characteristic crescent shape and ability to match the contours of the underlying anatomy. Such implants are available in a variety of sizes and may be further customized intraoperatively. Despite their widespread use, complications have been well documented including pain at the implantation site, infection, esthetic dissatisfaction, extrusion, and intrasinus migration. These complications often require evaluation via diagnostic imaging and subsequent surgical treatment. The most common complication is thought to be implant malpositioning, whereas infection, abscess, and intrasinus migration are less commonly seen [2]. With regard to alloplastic implants in the facial region, malar implants have been previously associated with the highest post-implantation infection rates at a rate of 2.67% [3]. Clinically, implant infections can manifest as cellulitis, abscess, draining sinuses, and osteomyelitis. High-resolution computed tomography (HRCT) and MRI are effective imaging modalities for diagnosing and detecting the extent of such complications along with intrasinus migration [4].

## Case presentation

**Figure 1:**
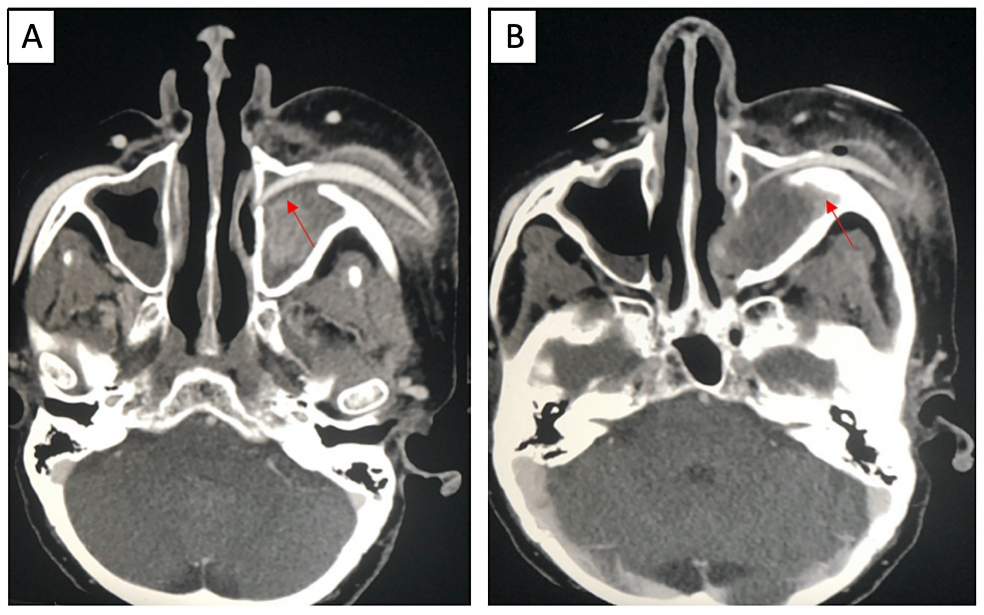
Enhanced CT, axial view. Enhanced CT displaying left-sided facial swelling, edema, and abscess. Penetration of the left malar implant into the left maxillary sinus and abut the lamina papyracea is noted (A) with left zygomatic bony erosion secondary to osteomyelitis (B).

**Figure 2:**
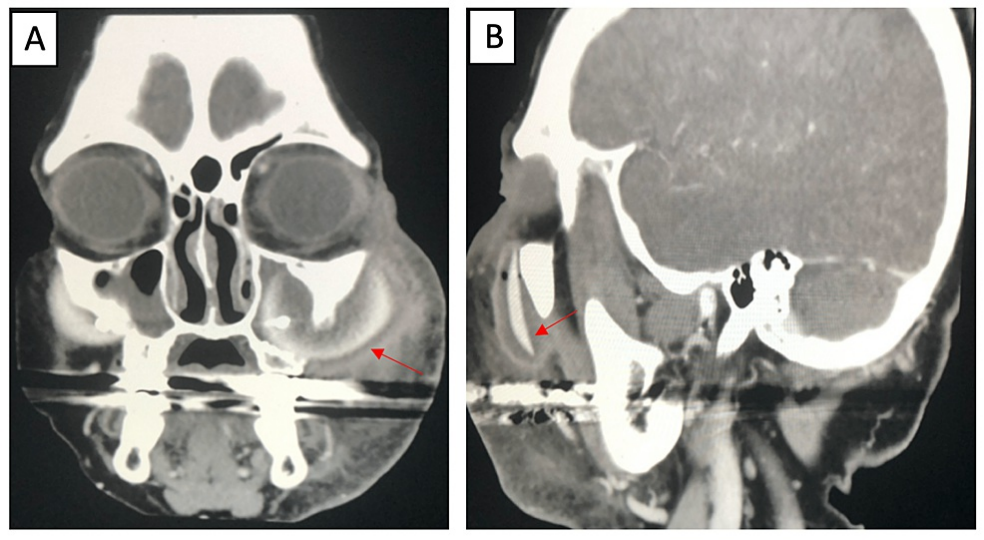
Enhanced CT in coronal (A) and sagittal (B) views. Enhanced CT displaying abscess formation in coronal view (A) around a left malar implant penetrating into the left maxillary sinus in sagittal view (B).

We present a 62-year-old female with a four-year history of bilateral silicone malar implants that presented with a five-day history of left-sided facial swelling and erythema. The patient reported having bilateral malar implants placed for cosmetic purposes. The patient denied any other past medical history, prior facial trauma, or previous complications with her implants. CT imaging was performed on initial evaluation, displaying a visualized left malar implant penetrating into the ipsilateral maxillary sinus (Figures 1A, 2B) with left-sided zygomatic bony erosion (Figure 1B). Abscess formation around the left maxillary sinus was also noted (Figure 2A).

The patient required endoscopic sinus surgery for removal of the offending foreign body. Systemic culture-directed antibiotic therapy was also initiated. The patient’s post-operative course was without complication following endoscopic removal.

## Discussion

Silicone implants in the facial region are useful for cosmetic purposes due to their volume enhancing effects. While most of these implants are without post-operative complications, maladies do occur with malpositioning being the most common [2]. Infection is also a possibility, particularly for implants in the malar region [3]. Although the customary treatment of implant-related infections previously looked towards prospective removal of the foreign body, conservative alternatives have more recently been favored. Such alternatives include wound debridement and localized delivery of targeted antibiotic therapy [5].

Intrasinus migration of malar implants, such as that reported in this case, is a rare development that complicates the use of the conservative alternatives described. Precedent reports have found foreign body removal as the more appropriate treatment in such cases [6]. Although previous case analyses have recognized this complication to occur in a delayed fashion, greater than one to two decades post-operative, this case described is unique in that the patient presented with intrasinus migration four years subsequent to implant placement [6-8].

The use of HRCT and MRI plays a fundamental role in determining the severity of this rare but serious complication. The increasing use of such implants displays the importance in educating radiologists on methods of recognizing and interpreting signs of infection via diagnostic imaging modalities. While the diagnosis of an infected facial silicone implant begins with a thorough history and physical exam, identification of such implants on imaging, along with localized soft tissue changes suggestive of post-implantation infection or implant migration, is of key importance in diagnosing and interpreting the extent of such complications.

## Conclusions

Silastic shell cosmetic cheek implants may result in complications such as infection, abscess and osteomyelitis, with symptoms including tenderness at the implantation site, swelling, warmth, and erythema. The uses of HRCT and MRI are advantageous imaging modalities that may aid clinicians in determining the severity and extent of malar implant complications.
